# Electrocardiography evaluation in type 1 diabetes mellitus, preliminary study

**DOI:** 10.1186/1687-9856-2015-S1-P6

**Published:** 2015-04-28

**Authors:** Nur Rochmah, Muhammad Faizi

**Affiliations:** 1 Faculty of Medicine-Airlangga University-dr Soetomo Hospital, Surabaya, East Java, Indonesia

## 

Cardiovascular complication should be evaluated in patient with T1DM. Ventricular instability (QT abnormalities) in Type 1 Diabetes Melitus (T1DM) is a risk factor for mortality. Prolongation of corrected QT interval (QTc) is accurate and the most sensitive test for the autonomic neuropathy whether QT interval dispersion (QTd) in arrhythmia is a predictor for mortality. This study is to evaluate arrhythmia, prolongation of QTc and QTd in T1DM children. Cross sectional study of children diagnosed T1DM in Soetomo Hospital; Surabaya during April 2013 to April 2014 was performed. The ECG was done in all of the patients. Arrhythmia, QTc and QTd were measured and analyzed with Paired t-test. There were 17 patients joint this study. Age was 8 to 15 years. There were 9 girls and 8 boys who suffered from 1 to 7 years of illness. There were 9/17 arrhythmia patients, 2/17 prolonged QTc patients, 4/17 borderline QTc patients, 1/17 with total AV block. The patient with total AV block was diagnosed DMT1 with ketoasidosis and acute pancreatitis. There was 1 patient without prolonged QTc although suffered from five times of DKA. The mean of QTc was 428.5 (SD 27.67) m.sec and QTd was 29.3 (SD 12.79) m.sec. There was significant differences between present of arrhythmia and QTd (P<0.01, 95% CI -35.01 to -20.85) and QTc (P<0.01, 95% CI -442.43 to -411.85). Arrhythmia and prolonged QTc was present in T1DM children under 10 years of illness. No one with prolonged QTd. Every arrhythmia should be taken into account in QTd to predict the mortality.

